# Supplementary material for: Cutoff CT value can identify upper gastrointestinal bleeding on postmortem CT: Development and validation study
Source: PLoS One. 2024 Jun 7;19(6):e0304993. doi: 10.1371/journal.pone.0304993 (PMC11161085; doi:10.1371/journal.pone.0304993)
Supplement: Attachment — Submitted filename: PMCT_UGIB_Response _240125.docx [file pone.0304993.s002.docx]

**Response to the Editors and the Reviewers**

Thank you very much for your letters of January 22^nd^ and 23^rd^, 2024, regarding our manuscript entitled, "Cutoff CT value can identify upper gastrointestinal bleeding on postmortem CT: development and validation study" (PONE-D-24-00950). We appreciate the reviewer's and the editor's comments.

*The editorial wrote (1/22/2024, 12:58):*

*“Based on our evaluation of your manuscript and the information you provided, we do not feel that your submission meets our ethical requirements for human subjects research submissions. PLOS ONE requires that research meets all applicable standards for the ethics of experimentation and research integrity (http://journals.plos.org/plosone/s/human-subjects-research). We reserve the right to reject any submission that does not meet our internal ethical standards, which in some cases are more stringent than local ethical standards.*

*We recognize that you obtained ethical approval from your local ethics committee after you began the study: your ethics approval document is dated 4 April 2023, while personal data were collected and accessed beginning in April 2021. For human subjects research, PLOS ONE requires that authors obtain ethics committee approval before beginning the study, and we do not accept retrospective ethical approval.*

*As a result of these concerns we cannot consider the manuscript for publication at PLOS ONE. I am sorry that we cannot be more positive on this occasion, but hope that you understand the reasons for this decision.”*

*The editorial wrote in response to our email that clarified the editor’s misunderstanding (01/23/2024 2:07):*

*“If you feel you can fully address the reviewers’ and the Academic Editor’s comments in a revised manuscript, you’re welcome to submit a new version of the manuscript for consideration as a new submission.*

*Please revise your manuscript to address the comments in the decision letter, submit your paper as a brand new submission, and do the following:*

*1. Refer to the previous submission (PONE-D-24-00950) in your cover letter*

*2. Upload a Response to Reviewers letter that includes a point by point response to each of the comments in your previous decision letter. This should be uploaded as a “Response to Reviewers” file type.*

*Again, I sympathize with your disappointment and I’m very sorry the outcome of the evaluation of your paper was not more positive. If you have any questions or need help with this process, don’t hesitate to reach out.”*

Our Response:

Our initial submission was remanded due to concerns over a potential ethics code violation. However, this was a result of a misunderstanding regarding the dates on our ethics review document. We have since clarified this with the editorial team and have been granted permission to resubmit.

To address the concerns, we wish to emphasize that the date of 4 April 2023 on our ethics review document only reflects the most recent update, specifically a change in our research team's composition. This date is not, as previously assumed, the original approval date of our ethics review. The ethics approval for our study was actually granted on 9 June 2008, as indicated by the ethics review number 2076. Our research commenced only after receiving this approval. The document submitted with the initial manuscript was an updated version, labeled 2076-(16), to denote the 16th year since the study began and its corresponding revision. Therefore, we attached the 1^st^ edition of the ethics review document labeled 2076, granted on 9 June 2008, and resubmitted the manuscript as a brand new paper.

We have uploaded a tracked version of the manuscript that includes no changes or tracks because we did not make any changes after the initial submission. Still, the editorial office indicated we uploaded a tracked version of the manuscript anyway.

We apologize for any confusion caused by not including the original ethics review document and appreciate your understanding in this matter. We are committed to the highest ethical standards in our research and look forward to the opportunity to contribute to the field through your esteemed journal.
