# Supplementary material for: Cutoff CT value can identify upper gastrointestinal bleeding on postmortem CT: Development and validation study
Source: PLoS One. 2024 Jun 7;19(6):e0304993. doi: 10.1371/journal.pone.0304993 (PMC11161085; doi:10.1371/journal.pone.0304993)
Supplement: Attachment — Submitted filename: PMCT_UGIB_Response _240321.docx [file pone.0304993.s004.docx]

**Response to the Editors and the Reviewers**

Thank you very much for your letters of March 8^th^, 2024, regarding our manuscript entitled, "Cutoff CT value can identify upper gastrointestinal bleeding on postmortem CT: development and validation study" (PONE-D-24-03120). We appreciate the reviewer's and the editor's comments.

Emails received from the editorial staff and reviewers are in blue italics, and our responses are in black after the ">" symbol.

*The editorial wrote (Mar/8/2024):*

*When submitting your revision, we need you to address these additional requirements.*

*1. Please ensure that your manuscript meets PLOS ONE's style requirements, including those for file naming. The PLOS ONE style templates can be found at*

*https://journals.plos.org/plosone/s/file?id=wjVg/PLOSOne_formatting_sample_main_body.pdf and*

[*https://journals.plos.org/plosone/s/file?id=ba62/PLOSOne_formatting_sample_title_authors_affiliations.pdf*](https://journals.plos.org/plosone/s/file?id=ba62/PLOSOne_formatting_sample_title_authors_affiliations.pdf)

> We have checked our manuscript style. We have added figure title in our main manuscript.

*2. Please expand the acronym “JSPS” (as indicated in your financial disclosure) so that it states the name of your funders in full.*

*This information should be included in your cover letter; we will change the online submission form on your behalf.*

> We have spelled out JSPS (Japan Society for the Promotion of Science) in the submission system and in the Cover letter.

*3. We note that you have indicated that there are restrictions to data sharing for this study. PLOS only allows data to be available upon request if there are legal or ethical restrictions on sharing data publicly. For more information on unacceptable data access restrictions, please see http://journals.plos.org/plosone/s/data-availability#loc-unacceptable-data-access-restrictions.*

*Before we proceed with your manuscript, please address the following prompts:*

*a) If there are ethical or legal restrictions on sharing a de-identified data set, please explain them in detail (e.g., data contain potentially identifying or sensitive patient information, data are owned by a third-party organization, etc.) and who has imposed them (e.g., a Research Ethics Committee or Institutional Review Board, etc.). Please also provide contact information for a data access committee, ethics committee, or other institutional body to which data requests may be sent.*

*b) If there are no restrictions, please upload the minimal anonymized data set necessary to replicate your study findings to a stable, public repository and provide us with the relevant URLs, DOIs, or accession numbers. For a list of recommended repositories, please see*

*https://journals.plos.org/plosone/s/recommended-repositories. You also have the option of uploading the data as Supporting Information files, but we would recommend depositing data directly to a data repository if possible.*

*We will update your Data Availability statement on your behalf to reflect the information you provide.*

> We have uploaded the minimal anonymized data set as Supporting Information files.

*4. Please review your reference list to ensure that it is complete and correct. If you have cited papers that have been retracted, please include the rationale for doing so in the manuscript text, or remove these references and replace them with relevant current references. Any changes to the reference list should be mentioned in the rebuttal letter that accompanies your revised manuscript. If you need to cite a retracted article, indicate the article’s retracted status in the References list and also include a citation and full reference for the retraction notice.*

> We have checked the references.

*Review Comments to the Author*

*Please use the space provided to explain your answers to the questions above. You may also include additional comments for the author, including concerns about dual publication, research ethics, or publication ethics. (Please upload your review as an attachment if it exceeds 20,000 characters)*

*Reviewer #1: Authors are using the terms CT and HU very frequently [for example, “mean CT value of the upper gastrointestinal contents was 48.2 Hounsfield units (HU)”]. Since this paper is going to be read by forensic people, who are not generally trained in radiology, the authors must explain these two concepts in simple terms. Rest of the paper appears okay to me.*

> We have added some introduction for CT and HU as follows:

“Computed tomography (CT) is a medical imaging technique that uses X-rays and computer processing to create detailed cross-sectional images of the body” (line 38),

“a standardized scale used in CT scanning to measure and compare the radiodensity of various substances: -1000 HU for air, 0 HU for water, and +1000 HU and beyond for dense materials like bone” (line 99).

*Reviewer #2: In total a very informative and practically significant article.*

*Statistical tests are ok, the charts and the figures are adequate.*

*Data collection method was ok, however, it is mentioned (line no. 19, 56) that it is retrospective case control study and at line No. 58-60 it is mentioned that “Written informed consent for the use of cadavers in our study was obtained from all families of the deceased participants” needs explanation.*

> We prospectively designed an overall outline of the research plan 16 years ago and collected data accordingly. While the current study was part of the original plan, the specific measurement items were ultimately determined retrospectively. As a result, we had initially described it as a retrospective study, but the overall design can be considered prospective. Therefore, we have removed the word "retrospective" in the Abstract, Materials and Methods, and Discussion.

*Reviewer #3: The article is a good research work in the field of Virtual Autopsy. This will help in upcoming centers of Virtual Autopsy for a more confirmatory diagnosis of Upper GI bleeding. The cutoff values mentioned will add to Medical literature.*

> Thank you very much. We appreciate your comment.

> In addition, we have corrected some inconsistencies in terminology and errors that I noticed during the review, leaving the changes tracked, but there are no changes to the content itself.

The typing error was amended: “derivation” changed to “validation” (line 172).

Inconsistencies in terminology was corrected in accordance with Materials and Methods: “inside” changed to “inside or close to” (lines 181 and 213).
