# Supplementary material for: Cutoff CT value can identify upper gastrointestinal bleeding on postmortem CT: Development and validation study
Source: PLoS One. 2024 Jun 7;19(6):e0304993. doi: 10.1371/journal.pone.0304993 (PMC11161085; doi:10.1371/journal.pone.0304993)
Supplement: Attachment — Submitted filename: Reviewer comment.pdf [file pone.0304993.s003.pdf]

In total a very informative and practically significant article.

Statistical tests are ok, the charts and the figures are adequate.

Data collection method was ok, *however, it is mentioned (line no. 19, 56) that it is retrospective case control study and at line No. 58-60 it is mentioned that "Written informed consent for the use of cadavers in our study was obtained from all families of the deceased participants" needs explanation*.
